# Expression of pannexin 1 and 2 in cortical lesions from intractable epilepsy patients with focal cortical dysplasia

**DOI:** 10.18632/oncotarget.14317

**Published:** 2016-12-28

**Authors:** Song Li, Zhenle Zang, Jiaojiang He, Xin Chen, Sixun Yu, Yuchun Pei, Zhi Hou, Ning An, Hui Yang, Chunqing Zhang, Shiyong Liu

**Affiliations:** ^1^ Epilepsy Research Center of PLA, Department of Neurosurgery, Xinqiao Hospital, Third Military Medical University, Chongqing 400037, China; ^2^ Department of Neurosurgery, General Hospital of the People's Liberation Army Lanzhou Military Region, Lanzhou, Gansu, 730050, China; ^3^ Department of Neurosurgery, General Hospital of the People's Liberation Army Chengdu Military Region, Chengdu, Sichuan, 610083, China

**Keywords:** focal cortical dysplasia, epileptogenesis, pathogenesis, pannexin 1, pannexin 2

## Abstract

Focal cortical dysplasia (FCD) is a major cause of intractable epilepsy in children however the mechanisms underlying the pathogenesis of FCD and FCD induced epilepsy remain unclear. Increasing evidence suggests that the large-pore ion channels, pannexin 1 (Panx1) and 2 (Panx2), are involved in epilepsy and brain development. In this study, we investigated the expression of Panx1 and Panx2 in surgical samples from patients with FCD type Ia (FCDIa), type IIa (FCDIIa), and type IIb (FCDIIb) and in age-matched autopsy control samples. We found Panx1 mRNA and protein levels were both increased in all these FCD samples. Immunohistochemical analyses revealed that Panx1 was mainly distributed in microcolumn neurons, dysmorphic neurons (DNs), balloon cells (BCs) and reactive astrocytes. Double-labeled staining showed that the Panx1-positive neurons were mostly glutamatergic DNs and occasionally GABAergic normal-appearing neurons. Importantly, the protein levels of Panx1 positively correlated with the frequency of seizures. Intriguingly, the Panx2 mRNA and protein levels were only upregulated in FCDIIb lesions and characteristically expressed on SOX2-positive multipotential BCs. Immunofluorescent experiments identified that Panx2-positive BCs mainly expressed the neuronal differentiation transcription factor MASH1 but not the immature glial marker vimentin. Taken together, our results established a potential role of the specific expression and cellular distribution patterns of Panx1 and Panx2 in FCD-associated epileptogenesis and pathogenesis.

## INTRODUCTION

Focal cortical dysplasia (FCD), which is characterized by cytoarchitectural abnormalities of the cerebral cortex, is well recognized as a major cause of intractable epilepsy in children [1–4]. Patients with FCD usually develop seizures at an earlier age than other surgically treated etiologies of epilepsy and should be considered candidates for surgery [1, 5]. The pathogenesis of FCD is thought to be mainly caused by embryonic developmental insults that result in the formation of dysplastic lesions with abnormal neuronal proliferation, migration and differentiation [2]. FCD is classified as three subtypes (FCD type I, FCD type II, and FCD type III) by the International League Against Epilepsy (ILAE) based on neuropathological examinations of surgical specimens, electroclinical presentations, imaging, and surgical outcomes. FCD type I (FCDI) and FCD type II (FCDII) refer to isolated lesions with cortical dyslamination, dysmorphic neurons (DNs) and balloon cells (BCs), whereas FCD Type III (FCDIII) is generally diagnosed in association with other epileptogenic lesions, such as hippocampal sclerosis, glioneuronal tumors and vascular malformations [6]. Although several studies have demonstrated the role of FCD induced epilepsy, the molecular mechanisms, such as gene expression and pathway activity that directly causes this type of seizure, remain largely unknown.

Pannexin (Panx) genes have been first described in the year 2000 and belong to the gap junction family because of the similarity between their structural features and those of gap junction proteins [7]. Three proteins coded by Panx genes have been identified: pannexin 1 (Panx1) and Panx2 are abundantly found in the central nervous system (CNS), while Panx3 is not [8]. Panx1 is broadly found in neurons and astrocytes, where it is a component of the large pore ion channel. It does not, however, constitute the gap junction. Several signaling molecules (e.g., ATP, Ca^2+^, arachidonic acid, glutamate, etc) are released through Panx1 channels. Panx1 utilizes specific mechanism to facilitate the release of signaling molecules. For instance, it activates infammasome for the release of pro-inflammatory cytokines, such as IL-1β [9, 10]. Recent studies in the CNS have suggested the Panx1 channels is important for certain physiological functions (e.g., synaptic plasticity, learning) [11] and its abnormalities account for several pathological processes (e.g., ischemia, tumorigenesis, epilepsy) [10]. Santiago et al. have presented direct evidence that the Panx1 channel could affect kainic acid-induced seizure activity by using a Panx1-knockout mouse model [12]. The Panx2 channel, which is less studied than the Panx1 channel, has been detected with low abundance in prenatal brain but much higher in postnatal brain [10]. It has been shown that Panx2 channel is involved in the differentiation of neural stem cells [13]. Considering the association of epilepsy and various developmental disorders with FCD, understanding the expression pattern and cellular localization of Panx1 and Panx2 in FCD could provide constructive insights into their potential roles in the epileptogenesis and pathogenesis associated with FCD.

In the present study, we analyzed the expression of Panx1 and Panx2 in surgically resected FCD samples via real-time quantitative PCR and western blotting. Moreover, we investigated the specific cellular distribution of Panx1 and Panx2 in FCDIa, FCDIIa, and FCDIIb samples in detail. We found that Panx1 expression was increased in the FCD lesions and specifically distributed in the abnormal cells, such as DNs and BCs. Intriguingly, Panx2 expression was only upregulated in FCDIIb lesions and characteristically expressed on multipotential BCs.

## RESULTS

### Comparison of clinical variables

The clinical variables of all the subjects who were enrolled in this study are summarized in Table 1. No significant differences in age were found between the autopsy controls and the FCD patients (p>0.05). Moreover, the histories of epilepsy duration and seizure frequency were similar among the FCDIa, FCDIIa and FCDIIb patients (p>0.05).

**Table 1:** Clinical variables of the subjects in FCD groups and autopsy controls

	CCx	FCDIa	FCDIIa	FCDIIb
Age (years)	6.18 ± 1.07	7.16 ± 0.87*	6.39 ± 0.93*	4.97 ± 0.74*
Epilepsy duration (years)	NA	3.90 ± 0.46	5.17± 0.78^#^	3.62 ± 0.62^#^
Seizure frequency (per month)	NA	52.55 ± 10.17	54.83 ± 17.49^†^	74.10 ± 23.66^†^

NA: No Available information.

* p > 0.05, FCD groups versus CCx;^#^ p > 0.05, † p > 0.05, FCDIIa FCDIIb versus FCDIa. One way ANOVA.

### Quantitative real-time PCR and western blotting analysis of Panx1 and Panx2

**Figure 1:**
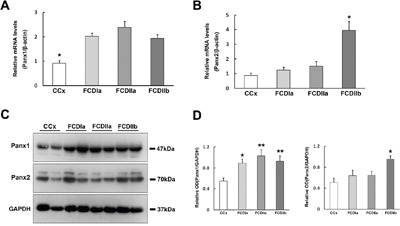
Expression of Panx1 and Panx2 in FCD and CCx **A**. Real-time PCR analysis of Panx1 mRNA expression in CCx (n = 8), FCDIa (n = 8), FCDIIa (n = 8) and FCDIIb (n = 8). * p < 0.05, CCx versus FCDIa, FCDIIa and FCDIIb. **B**. Panx2 mRNA expression in CCx and FCD groups. * p < 0.05, FCDIIb versus CCx, FCDIa and FCDIIa. Panx1 and Panx2 mRNA expression for each sample was normalized to β-actin. **C**. Representative immunoblot bands of Panx1 and Panx2 protein in total homogenates from CCx samples and FCD lesions. Molecular weight detected with Panx1 and Panx2 is shown at the expected bands of 47 kDa and 70 kDa. **D**. Densitometric analyses of Western blots. Values (Relative OD) are expressed as mean±SEM. * p < 0.05, ** p < 0.01. One way ANOVA.

The expression levels of Panx1 and Panx2 mRNA in FCD lesions and autopsy controls were determined via quantitative real-time PCR. The results show that the Panx1 and Panx2 transcripts were detected in all brain tissues and the Panx1 transcript levels in FCDIa, FCDIIa and FCDIIb cortical lesions were significantly higher than the levels in the control cortex (CCx) samples (Figure 1A). However, the Panx2 transcript levels were similar in the CCx, FCDIa and FCDIIa samples but were significantly increased in the FCDIIb samples (Figure 1B).

In the western blotting analysis of tissue homogenates, Panx1 and Panx2 were detected as bands of approximately 47 kd and 70 kd, respectively (Figure 1C). A statistical analysis showed that the Panx1 protein levels in the FCDIa, FCDIIa, and FCDIIb cortical lesions were significantly higher than the levels in the CCx samples (Figure 1D, left panel). In accordance with the PCR findings, the expression of the Panx2 protein was prominently increased in the FCDIIb samples versus its expression in the CCx, FCDIa and FCDIIa samples (Figure 1D, right panel).

### Panx1 immunoreactivity (IR) in the FCD lesions

**Figure 2:**
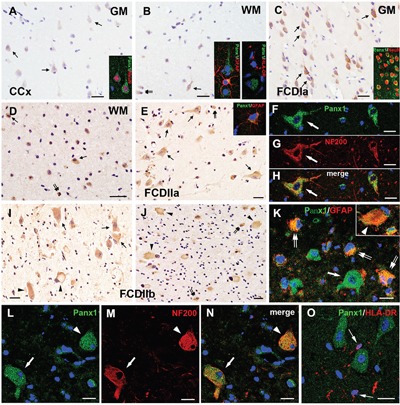
Panx1 immunoreactivity (IR) in cortical lesions of FCD **A-B**. Weak to moderate Panx1 IR in neurons (arrows in **A**), glial cells (double arrows in **B**) in gray matter (GM) and white matter (WM) of CCx. Insert in **A** refers NeuN positive neuron colabeled with Panx1. Insert in **B** refers GFAP positive astrocyte, not HLA-DR positive microglia colabeled with Panx1. **C-D**. Panx1 IR in FCDIa. Moderate to strong Panx1 IR in neurons (arrows in **C**), including in NeuN positive microcolumns (insert in **C**). Strong Panx1 IR in ectopic neurons (arrows in **D**) and glial cells (double arrows in **D**). **E**. Panx1 IR in FCDIIa. Moderate to strong Panx1 IR in DNs (arrows) and gliall cells (double arrows). Insert in **E** refers GFAP positive astrocyte colabeled with Panx1. **F-H**. Confocal images showing colocalization of Panx1 (green) with NF200 (red) in DN (arrow) in FCDIIa. **I-J**. Panx1 IR in FCDIIb. Moderate to strong Panx1 IR in DNs (arrows), BCs (arrowheads) and glial cells (double arrows). **K**. Merged images showing colocalization of Panx1 (green) with GFAP (red) in reactive astrocytes (double arrows) and BC (arrowhead, insert in **K**), but not in DN (arrow). **L-N**. Confocal images showing colocalization of Panx1 (green) with NF200 (red) in DN (arrow) and BC (arrowhead). **O**. Double labeling staining shows the HLA-DR (red) positive microglias (arrows) don't colocalize with Panx1 (green). 5-μm paraffin-embedded sections are counterstained with hematoxylin **(A-E, I, J)** or DAPI **(F-H, K-O)**. Scale bars = **(A-E, I, J)** 30 μm, **(F-H, K-O)** 20 μm.

As described previously [8, 14], weak to moderate Panx1 immunoreactivity (IR) was observed in NeuN positive neurons, and Panx1 IR-positive glial cells were occasionally observed in the white matter (WM) of the CCx tissues (Figure 2A, 2B). These glial cells were confirmed as GFAP positive astrocytes, but not HLA-DR positive microglia (Figure 2B, insert). Moderate to strong Panx1 staining was detected in neurons (Figure 2C), including in NeuN-positive microcolumns (Figure 2C, insert) and ectopic neurons (Figure 2D) in FCDIa cortical lesions. In addition, moderate staining was observed in glial cells (Figure 2D). The staining scores suggested a higher expression of Panx1 in the FCDIa samples than that in the CCx samples (Table 2). Moreover, there was substantially increased reactivity per neuron and astrocyte in the FCDIa lesions (Table 3).

**Table 2:** Staining scores of Panx-immunopositive cells in CCx and FCD groups

	CCx(n = 10)	FCDIa(n = 11)	FCDIIa(n = 12)	FCDIIb(n = 10)
Panx1	0.93 ± 0.15	2.01 ± 0.24*	2.22 ± 0.28**	2.08 ± 0.21*
Panx2	0.67 ± 0.19	0.75 ± 0.27	0.82 ± 0.23	1.76 ± 0.30^#^

* p < 0.05, FCDIa, FCDIIb versus CCx.

** p < 0.01, FCDIIa versus CCx.

# p < 0.05, FCDIIb versus FCDIa, FCDIIa, CCx. One way ANOVA.

**Table 3:** Cell-type distribution of Panx1 in CCx and FCD groups (% of cases with immunoreactive cells)

	CCx (n = 10)								FCDIa (n = 11)								FCDIIa (n = 12)								FCDIIb (n = 10)											
	neurons				astrocytes				neurons				astrocytes				neurons				astrocytes				neurons				astrocytes				BCs			
Intensity	-	+	++	+++	-	+	++	+++	-	+	++	+++	-	+	++	+++	-	+	++	+++	-	+	++	+++	-	+	++	+++	-	+	++	+++	-	+	++	+++
Panx1	0	47	53	0	30	52	18	0	0	17	30	53	12	26	47	15	0	10	35	55	5	21	36	38	0	12	37	51	3	14	42	41	13	15	42	30

Staining: ―, absent (0); +, weak (1); ++, moderate (2); +++, strong staining.

In FCDIIa cortical specimens, moderate to strong staining for Panx1 was detected in neurons, espically in DNs (93±4.1%, n=628, from 12 samples) (Figure 2E), and strong staining was also detected in GFAP-positive astrocytes (Figure 2E, insert) (Table 3). The staining scores indicated an upregulation of Panx1 expression in FCDIIa lesions compared with the one in CCx samples (Table 2). Double-labeled immunofluorescent staining results indicated that Panx1-positive DNs could be labeled with NF200, a neuronal marker (Figure 2F–2H).

In FCD IIb cortical tissues, moderate to strong staining for Panx1 was detected in nerurons, and especially in DNs (96±3.4%, n=513, from 10 samples), balloon cells (BCs) (72%±7.1%, n=298, from 10 samples), as well as in glia cells (Figure 2I, 2J) (Table 3). The intensity scores of Panx1 IR in the FCDIIb cortical lesions were significantly higher than those in the CCx samples (Table 2). Further immunofluorescent studies demonstrated that Panx1 was co-located with GFAP in BCs and reactive astrocytes (Figure 2K). Moreover, the Panx1 positive DNs and BCs were co-labelled with NF200 (Figure 2L-2N), but not with the microglia marker HLA-DR (Figure 2O).

**Figure 3:**
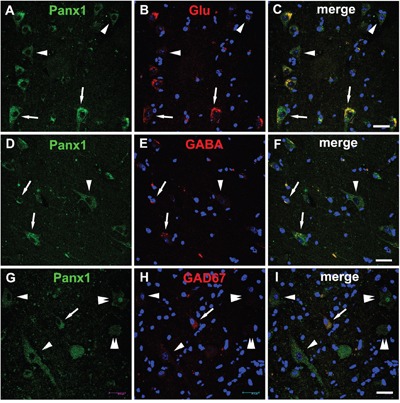
Double immunofluorescent staining of Panx1 in neurons in FCD **A-C**. Representative confocal images show that strong Panx1 positive (green) DNs (arrows) colocalize with glutamate (Glu, red), and weak Panx1 positive (green) neurons (arrowheads) don't colocalize with Glu (red). **D-F**. Merged images show colocalization of Panx1 (green) with γ-aminobutyric acid (GABA, red) in the normal-appearing neurons (arrows), but not in the DN (arrowhead). **G-I**. Double labeling staining shows colocalization of Panx1 (green) with glutamic acid decarboxylase 67 (GAD67, red) in normal-appearing neuron (arrow), but not in the DNs (arrowheads) and BCs (double arrowheads). 5-μm paraffin-embedded sections are counterstained with DAPI. Scale bars = 30 μm.

Histological studies have shown that the Panx1 protein was presented in the excitatory pyramidal cells and γ-aminobutyric acid (GABA)ergic interneurons in the neocortex and hippocampus [8, 15]. To further investigate the cellular distribution of Panx1, double-labeled immunofluorescence assays were performed. These assays demonstrated that in FCD lesions, DNs with strong Panx1 IR were glutamate positive, while neurons with weak Panx1 IR were mostly glutamate negative and morphologically normal (Figure 3A-3C). In addition, we found that the Panx1-positive and morphologically normal neurons, which are not typical DNs, expressed GABA (Figure 3D–3F). Further examinations revealed the colocalization of Panx1 with glutamic acid decarboxylase 67 (GAD67) in normal-appearing neuron but not in the DNs or BCs (Figure 3G–3I).

### Panx2 immunoreactivity in the FCD lesions

**Figure 4:**
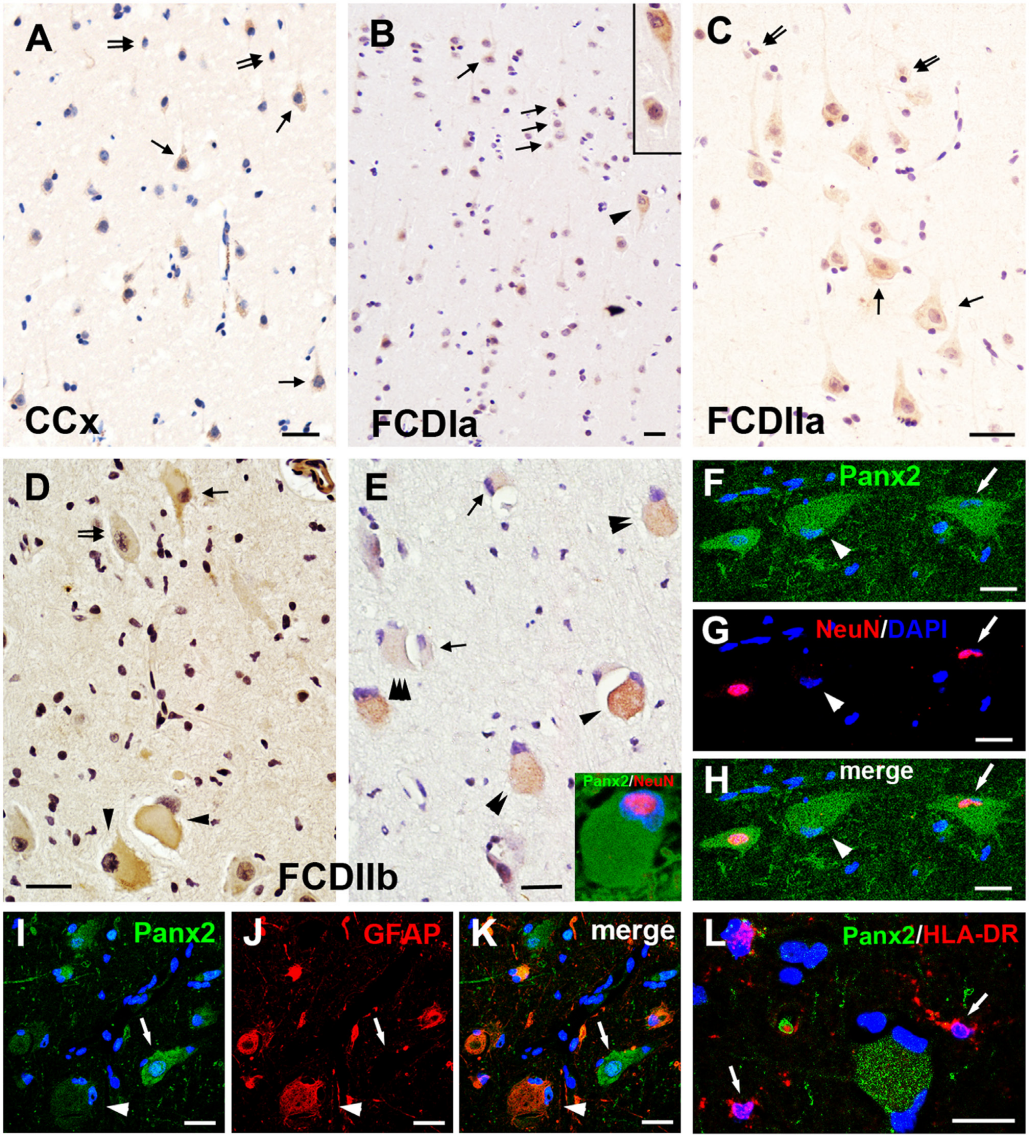
Panx2 immunoreactivity (IR) in cortical lesions of FCD **A**. Weak to moderate Panx2 IR in neurons (arrows) and glial cells (double arrows) in gray matter of CCx. **B**. Panx2 IR in FCDIa. Weak to moderate Panx2 IR in neurons (arrows), including in microcolumns and HNs (arrowhead, insert in **B**). **C**. Panx2 IR in FCDIIa. Weak to moderate Panx2 IR in DNs (arrows) and glial cells (double arrows). **D-E**. Panx2 IR in FCDIIb. Moderate to strong Panx2 IR in BCs (arrowheads in **D**). Weak (double arrows in **D**) and strong (arrow in **D**) Panx2 IR in DNs. Clusters of weak (triple arrowheads in **E**), moderate (double arrowheads in **E**) and strong (arrowhead in **E**) Panx2 IR positive BCs in FCDIIb. Weak to moderate Panx2 IR in glial cells (arrows in **E**). **F-H**. Double labeling staining shows colocalization of Panx2 (green) with NeuN (red) in DN (arrow) in FCDIIb. Some Panx2 positive BC colocalize with NeuN ( insert in **E**), and some are NeuN negative (arrowhead). **I-J**. Representative confocal images show the GFAP (red) positive BC (arrowhead) and reactive astrocytes (doublearrows) colocalize with Panx2 (green). GFAP negative DN (arrow) with stronger Panx2 IR than the GFAP positive cells. **L**. Merged images shows the HLA-DR (red) positive microglias (arrows) don't colocalize with Panx2 (green). 5-μm paraffin-embedded sections are counterstained with hematoxylin **(A-E)** or DAPI **(F-L)**. Scale bars = **(A-E)** 30 μm, **(F-L)** 20 μm.

Weak to moderate Panx2 IR was observed in neurons and occasionally in glial cells of the CCx tissues (Figure 4A). In FCDIa cortical samples, weak to moderate Panx2 staining was detected on neurons, including the microcolumns and hypertrophic neurons (HNs) (Figure 4B). Similarly, weak to moderate Panx2 staining was detected in DNs and glial cells in FCDIIa samples (Figure 4C). In accordance with the PCR and western blotting results, the staining scores suggested that there were no significant differences in the expression of Panx2 among the FCDIa, FCDIIa and CCx group (Table 2).

In FCD IIb cortical tissues, weak and occasionally strong Panx2 staining was detected on DNs and glial cells. However, moderate to strong staining for Panx2 was detected on 60%±8.5% of BCs (n=314, from 10 samples) (Figure 4D, 4E). The staining scores demonstrated an upregulation of Panx2 expression in the FCDIIb lesions compared with its expression in the FCDIa, FCDIIa and CCx samples (Table 2). Double-labeled immunofluorescent results indicated that the Panx2-positive DNs and a portion of Panx2-postive BCs could be labeled with NeuN (Figure 4E insert, 4F-4H). Furthermore, the Panx2 co-located with GFAP in BCs, but not in DNs (Figure 4I-4K). Moreover, the HLA-DR positive microglia did not express Panx2 (Figure 4L).

**Figure 5:**
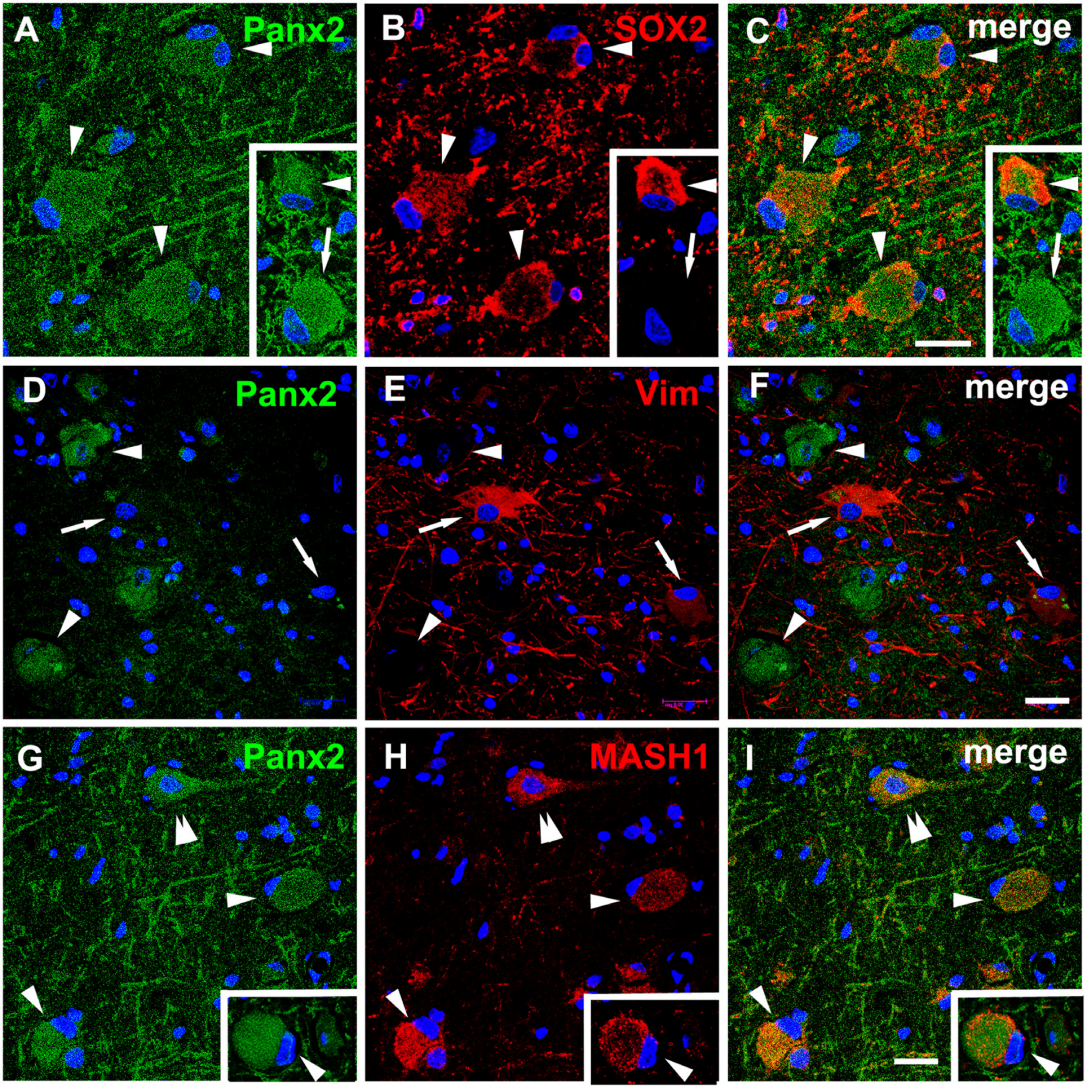
Double immunofluorescent staining of Panx2 in BCs in FCDIIb **A-C**. Representative confocal images show some Panx2 positive (green) BCs (arrowheads) colocalize with SOX2 (red), and some are SOX2 negative (arrow, insert). **D-F**. Double labeling staining shows the Panx2 (green, arrowheads) don't colocalize with vimentin (vim, red, arrows) in BCs. **G-I**. Merged images show Panx2 (green) colocalize with MASH1 (red) in pyramidal neuron (double arrowheads) and BCs (arrowheads). 5-μm paraffin-embedded sections are counterstained with DAPI. Scale bars = 30 μm.

Considering the abnormal differentiation of the BCs and the specific cellular distribution of Panx2 in these cells, we investigated the correlation between the Panx2 expression and the differentiation status of the BCs. Double-labeled immunofluorescent experiments confirmed that the Panx2 was colocalized with SOX2 (a marker of neural stem cells commonly expressed by BCs [16]) in BCs (Figure 5A–5C). Further double-labeled staining showed that the Panx2 positive BCs did not express vimentin (a marker of immature glial cells) (Figure 5D-F), but expressed MASH1 (a basic helix-loop-helix transcription factor involved in the determination of neuronal precursors) (Figure 5G–5I).

### Correlation between the Panx1 protein levels and the clinical variables of FCD

**Figure 6:**
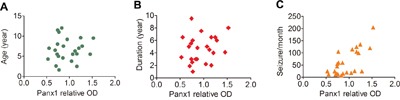
Correlation between the protein levels of Panx1 and different clinical variables in FCD **A, B**. Scatter plot showing no significant correlation between the protein levels (relative optical density [OD]) of Panx1 and age at surgery (r = 0.135, P = 0.531), or duration of epilepsy (r = 0.182, P = 0.394). **C**. Scatter plot showing the significant positive correlation between the protein levels of Panx1 and seizure frequency (seizures per month), (r = 0.627. P = 0.001).

Correlations between Panx1 protein levels and different clinical variables (age at surgery, epilepsy duration, seizure frequency) of all the FCD patients were assessed. There were no significant correlation between the protein levels of Panx1 and age at surgery (Figure 6A; r = 0.135, P = 0.531), or duration of epilepsy (Figure 6B; r = 0.182, P = 0.394). However, we found that the protein levels of Panx1 in FCD positively correlated with the frequency of seizures before surgery (Figure 6C; r = 0.627. P = 0.001).

## DISCUSSION

In the present study, we observed that the expression of Panx1 was greater in FCDIa, FCDIIa and FCDIIb epileptic lesions than that in control CCx samples at both mRNA and protein levels. Further immunohistochemical (IHC) experiments revealed that Panx1 was highly expressed in neuronal microcolumns, DNs, BCs, and reactive astrocytes, and was characteristically co-localized with glutamatergic and GABAergic neural markers in FCD lesions. Moreover, the protein levels of Panx1 positively correlated with the frequency of seizures. Intriguingly, compared with those in CCx samples, the mRNA and protein levels of Panx2 were only upregulated in FCDIIb lesions, and not in FCDIa and FCDIIa lesions. Moreover, in FCDIIb lesions, Panx2 protein was primarily identified in BCs, where it is co-localized with SOX2 and MASH1, but not with vimentin. Based on these results, we concluded that Panx1 might participate in the epileptogenesis with FCD and Panx2 might be involved in the pathogenesis of FCDIIb lesions.

### The role of Panx1 in epilepsy associated with FCD

Increasing experimental and clinical evidence indicates that Panx1 overexpression may be associated with epilepsy. The expression of Panx1 was found to be up-regulated in the brain slices under Co^2+^-induced hippocampal seizure activity [17]. In animal models of temporal lobe epilepsy, Santiago et al. found that the knockout of Panx1 shortened the duration of kainic acid-induced status epilepticus by decreasing the extracellular ATP levels [12]. Meanwhile, the protein level of Panx1 has been found to be upregulated in surgically removed brain tissue of patients with temporal lobe epilepsy [18]. Cepeda et al found that the expression of Panx1 was up-regulated in Rasmussen encephalitis, which is an important cause of intractable epilepsy, and blockade of Panx1 channel could reduce the epileptogenesis [19]. Similar to these previous findings, we found that the expression of Panx1 in FCDIa, FCDIIa and FCDIIb epileptic lesions was increased relative to its expression in CCx samples at both mRNA and protein levels via quantitative real-time PCR and western blotting analysis. Importantly, the protein levels of Panx1 positively correlated with the frequency of seizures, which suggested a potential role of Panx1 in FCD induced epilepsy.

Histological studies have confirmed that the Panx1 protein was located at the soma and asymmetric postsynaptic sites in hippocampal and cortical principal neurons [20], which supported us the structural basis of Panx1 in the synaptic function. Upon the exposure to anoxia or ischemia, Panx1 channels can be activated, and the opening of Panx1 leads to a sustained neuronal depolarization [21]. Furthermore, the opening of Panx1 channels triggered by NMDA receptor stimulation and its contribution to epileptiform seizure activity has been well-recognized in studies by Thompson et al [22, 23]. Blockade of Panx1 channel could reduce the epileptiform activity of cultured pyramidal neurons from patients with Rasmussen encephalitis [19]. Our IHC studies detected Panx1 expression at characteristically high levels in neuronal microcolumns and DNs, which may be the intrinsic “pacemakers” that initiate and drive the epileptiform activity in FCD [24, 25]. Likewise, the double-labeled staining demonstrated that Panx1 was mostly co-localized with glutamate on DNs and with GABA and GAD67 on normal-appearing neurons, suggesting that the activation of Panx1 may lead to a change in the excitatory/inhibitory balance of neuronal circuits in the epileptic zone. Therefore, we concluded that Panx1 might be associated with cell-induced epilepsy in FCD.

Panx1 is a large-pore ion channel expressed in astrocytes, with its most well-known channel function being the release of ATP [9]. It's well-recognized that ATP is released in high amounts into the extracellular space as a gliotransmitter, potently modulating the generation of seizures, inflammation and seizure-induced brain damage [26]. High extracellular potassium concentrations, a potential cause of seizures, opens Panx1 channels and leads to inflammasome activation in astrocytes [27]. Concomitantly, Panx1 promotes the release of pro-inflammatory cytokine such as IL-1β by activating the inflammasome [28]. Moreover, the Panx1 channels may have a role in mediating glutamate release from astrocytes [29]. Our IHC studies found Panx1 at characteristically high levels in GFAP-positive BCs and reactive astrocytes, but not in microglia. Accumulating data indicated that glial cells in the cortical lesions of FCD may play critical roles in epileptogenesis. Our previous studies [30, 31] and those from other groups [32, 33] have provided evidence suggesting that the glial derived inflammatory cytokines (e.g. IL-6, IL-17, IL-1β) contribute to the high epileptogenicity of FCD lesions. It is therefore possible that glial expressed Panx1 channels may be involved in the epileptogenic capacity of FCD.

### The role of Panx2 in the pathogenesis of FCDIIb

According to the classification of FCD defined by the ILAE, FCDIIb lesions share the most typical histological manifestation characterized by the balloon cells (BCs) compared with the other variants [6]. These histopathological differences suggest that there are distinct mechanistic factors leading to their formation. Vogt et al have confirmed that the level of Panx2 mRNA was low in the prenatal period and increased substantially during postnatal development [15], which supporting a potential role of Panx2 in the development of the CNS. In our research, there were no significant differences in Panx2 expression among the CCx, FCDIa and FCDIIa groups. Interestingly, the expression of Panx2 was significantly elevated at both the mRNA and protein levels in the FCDIIb lesions. To our knowledge, there were few reports about the role of Panx2 in epilepsy. Considering the ages, histories of epilepsy and seizure frequencies were similar among patients with the three types FCD, the specific expression patterns indicated a potential role of Panx2 in FCDIIb.

It has been widely accepted that the formation of FCDIIb lesions is a result of embryonic developmental disorders [34]. Accumulating molecular pathological evidence demonstrates that the FCDIIb lesions are characterized by progenitor cell markers and share abnormalities with delayed neural cells maturation, which distinguishes it from the other lesion types [16, 35]. Several studies have found that the BCs express stem cell markers (e.g., CD133, SOX2, doublecortin) [16, 36, 37], neural progenitor cell markers (e.g., CRMP4, MASH1) [37] and immature glial cell markers (e.g., vimentin) [38]. Our IHC study revealed that Panx2 was characteristically highly expressed on the BCs. Consistent with previous reports about the multipotential differentiation status of BCs, we found that Panx2 was colocalized with SOX2 on BCs. Further double-labeled immunofluorescent experiments confirmed Panx2-positive BCs expressed the neuronal differentiation transcription factor MASH1, but not the immature glial marker vimentin. Panx2 protein has been found expressed by multipotential progenitor cells of the hippocampus and knockdown of Panx2 significantly accelerates the rate of neuronal differentiation, which suggests a inhibitory role of Panx2 in neural stem cell differentiation [13]. Taken together, the upregluation of Panx2 in FCDIIb lesions and the specific distribution of Panx2 on BCs support our hypothesis that Panx2 might participate in the pathogenesis of FCDIIb, although further functional experiments are needed to thoroughly elucidate the molecular mechanism.

In conclusion, to our knowledge, this is the first demonstration of an association between the overexpression of Panx1 and the epileptogenic nature of FCD. Our IHC study further displayed that Panx1 was specifically distributed in the abnormal cells, suggesting that Panx1 might participate in the processes of these cells induced epilepsy. Moreover, another Panx family member, Panx2 was only upregulated in FCDIIb lesions, and was characteristically expressed on BCs, which indicated Panx2 might be involved in the pathogenesis of FCDIIb lesions. Based on the findings of the present descriptive study, the underlying mechanisms by which Panx1 and 2 regulate the epileptogenesis and pathogenesis of FCD and whether targeting the Panx1 or Panx2 may be beneficial as therapeutic strategies in patients will be investigated in future studies

## MATERIALS AND METHODS

### Subjects

The cases included in this study were obtained from the Department of Neurosurgery of the Xinqiao Hospital (Third Military Medical University, Chongqing, China). All procedures and experiments were guided under the guidelines approved by the Ethics Committee of the Third Military Medical University. All the participants in both groups voluntarily joined this study with informed consents. The human brain tissues were obtained and used in a manner compliant with the Declaration of Helsinki.

Total of 33 patients samples with medically intractable epilepsy were examined, including FCDIa (n=11), FCDIIa (n=12) and FCDIIb (n=10), and the detailed clinical data (e.g., age at surgery, cortical lesion location, postoperative seizure outcome) are listed in Supplementary Table 1. FCD grading was according to the classification systems published by the International League Against Epilepsy (ILAE) [6]. Ten age-matched patients were obtained from autopsies as control cortex samples (CCx) which without history of seizures or other neurologic disease. All autopsies were performed within 6 h of death. Within this post-mortem interval, it is well-documented that most proteins are stable and therefore well preserved [39]. The detailed information for the control patients is listed in Supplementary Table 2. Two neuropathologists reviewed the control cases, and both gross and microscopic examinations revealed no abnormalities.

### Tissue preparation

All brain samples used in this study obtained from surgery were immediately divided into two parts. One part was fixed in 10 % buffered formalin 24-36 hours, then dehydrated and embedded in paraffin, Paraffin-embedded tissue was sectioned at 5 μm for the subsequent histological and immunohistochemical staining. The remaining samples were immediately placed in chilled tubes, and were snap-frozen in liquid N2 and stored at -80 °C until used for real-time PCR and western blotting analysis.

### Quantitative real-time PCR analysis

Extraction of total RNA[CCx(n=8), FCDIa(n=8), FCDIIa(n=8) and FCD IIb (n=8)]from the brain tissues used RNAiso Plus (TaKaRa, Japan), and then reverse transcribed into cDNA used PrimeScript™ RT reagent Kit with gDNA Eraser (TaKaRa). The relative quantification of Panx1 and Panx2 mRNA levels compared with the internal control gene β-actin were calculated according to the 2 (-ΔΔC(T)) method. For quantitative real-time PCR, gene specific primers were designed as follows: ACTIN: 5'-GCACCACACCTTC TACAATGAGC-3'and 5'-TAGCACAGCCTGGATAGCAACG-3'. Panx1: 5'-CATCTTCCAGTTGCTCAGTGTC-3'and 5'- GCCAAGGTTTGTCAGGAGTAG -3'. Panx2: 5'-ACCTGTTCGAGAAGTACCTGGAG-3' and 5'-GTTGACGAGGATGATGAGGTTC-3'. The amplification system was followed according to the instruction of PrimeScriptTM RT reagent Kit with gDNA Eraser (Perfect Real Time) (TaKaRa) on CFX Connect™ Real-Time PCR Detection System (Bio-Rad). The PCR thermocycling conditions were as follows: 95°C for 10min, followed by 35 cycles of 95°C for 30 s, 56°C for 30 s, and 72°C for 30s. All of the samples were run in triplicate, and the relative quantification of each target gene expression was performed twice.

### Western blotting

We extracted total protein from frozen brain tissues which contained histologically normal (n=10), FCDIa(n=8), FCDIIa(n=8) and FCD IIb (n=8) cortices for immunoblot analyses used BestBio Kit at 4°C, then the concentration was determined using protein assay solution(Boster, Wu Han, China). The GADPH was used as internal reference. Equivalent amounts of proteins were separated by 10% sodium dodecyl sulfate-polyacrylamide gel electrophoretic gel (SDS-PAGE gel) and then transferred to polyvinylidene fluoride membranes by wet transfer. After blocked with 5% milk for 1h, the membrane was incubated overnight at 4°C with anti-Panx1 antibody (rabbit polyclonal, 1:500, Sigma, St Louis, MO, USA, Cat No.AV42783), anti-Panx2 antibody (rabbit polyclonal, 1:500, Sigma, Cat No.AV42778) and rabbit anti-GAPDH monoclonal (1:400, Millipore, Temecula, CA, USA, Cat No.AB2302), Then incubation with horseradish peroxide-conjugated anti-rabbit secondary antibodies (1:2000, Biotime, Nanjing, China) for 1 h at 37°C incubator, The membrane signals using enhanced chemiluminescence were visualized.

For immunoblotting analysis, densitometric analysis was performed using Image-Pro Plus software (Media Cybernetics, Silver Spring, MD). The optical densities (OD) of each protein band were calculated relative to the OD value of the reference complementary protein GAPDH.

### Histology and immunohistochemistry

5-μm-thick paraffin sections were mounted on the polylysine-coated slides. Two slices in each paraffin block were routinely stained with hematoxylin and eosin (H&E), and the consecutive serial sections were used for immunohistochemistry (IHC). The paraffin-embedded sections were deparaffinized, rehydrated, and incubated for 30 min in 0.3% H_2_O_2_ diluted in methanol to quench the endogenous peroxidase activity. All the samples were placed into phosphate buffered saline (0.01 M, pH 7.3) and heated in a microwave oven for the antigen retrieval for 20 minutes. Then sections were incubated for 1 h at room temperature followed by incubation at 4°C overnight with primary rabbit anti-human Panx1 (1:200, Sigma) and rabbit anti-human Panx2(1:200, Sigma). After 3 rinses, sections were then incubated with the secondary goat anti-rabbit immunoglobulin conjugated to peroxidase-labeled dextran polymer [EnVision+ System-HRP; Boster] for 60 minutes at 37°C. Immunoreactions were visualized with 3,3-diaminobenzidine (Boster). Sections were counterstained with hematoxylin. Negative control sections went through the same protocol with the exception of the primary antibody. The Leica DMIRB microscope (Leica, Nussloch, Germany) was used to capture the images of the tissue sections.

For double immunofluorescent staining, sections were incubated for 1 h at room temperature followed by incubation at 4°C overnight with primary anti-Panx1/2 combined with anti-NeuN (mouse monoclonal, 1:200; Millipore, Cat No.MAB377), or anti-GFAP (mouse monoclonal, 1:500; Sigma, Cat No.C9205), or anti-NF200 (mouse monoclonal, 1:100; Boster, Cat No.BM0100), or anti-HLA-DR (mouse monoclonal, 1:100; Sigma, Cat No.SAB4700731), or anti-Glutamate (mouse monoclonal, 1:1500; Sigma, Cat No.G9282), or anti-γ aminobutyric acid (GABA) (mouse monoclonal, 1:500; Sigma, Cat No.A0310), or anti-GAD67 (mouse monoclonal, 1:500; Sigma, Cat No.G5419), or anti-vimentin (mouse monoclonal, 1:100; Boster, Cat No.BM0135), or anti-Sox2 (mouse monoclonal, 1:200; Millipore, Cat No.MAB4343), or anti-MASH1 (mouse monoclonal, 1:200; Millipore, Cat No.MABD64). After 3 washes, the sections were incubated with a mixture of FITC-conjugated goat anti-rabbit IgG (1:200; Zhongshan Goldenbridge Biotechnology Co) and Alexa Fluor 594 goat anti-mouse IgG (1:500, Invitrogen, Eugene, OR, USA) for 1 hour at 37°C. And then, DAPI (10μg/ml, Beyotime) was used for the counterstaining of cell nuclei. Negative controls were carried out without primary antibody. Fluorescent sections were observed and photographed with a confocal laser scanning microscope (TCS-TIV; Leica, Nussloch, Germany).

### Evaluation of immunostaing and cell counting

Panx1and Panx2 immunoreactivity (IR) were evaluated as our previously reported, using a Leica microscope and examining in each section for a total microscopic area of 781.250 μm^2^ (200 high-power non-overlapping fields of 0.0625 × 0.0625 mm width, using a square grid inserted into the eyepiece). The intensity of staining was evaluated using a semi-quantitative three-point scale where IR was defined as: ―, absent (0); +, weak (1); ++, moderate (2); +++, strong staining (3). The score represents the predominant staining intensity in each section and was calculated as the average from the selected fields. Moreover, we calculated the labeling index (LI) of Panx1/2 positive abnormal cells in FCD lesions. Only these cells, which could be clearly identified as typical abnormal cells were included in this analysis. The LI was difined as the ratio of immunolabeled cells related to the entire cell population of interest.

### Data analysis and statistics

Data are expressed as mean ± SEM. Statistical analyses were performed with SPSS for Windows (SPSS 13.0, SPSS Inc., Chicago, IL, USA). Means were compared using independent-samples t-test for two groups, and one way ANOVA for more than two groups. p < 0.05 was considered significant. The correlations between variables were evaluated by the Spearman rank correlation test.

## SUPPLEMENTARY MATERIALS TABLES




