# Supplementary material for: Expression of pannexin 1 and 2 in cortical lesions from intractable epilepsy patients with focal cortical dysplasia
Source: Oncotarget. 2016 Dec 28;8(4):6883–95. doi: 10.18632/oncotarget.14317 (PMC5351677; doi:10.18632/oncotarget.14317)
Supplement: Supplementary file 1 [file oncotarget-08-6883-s001.pdf]

Expression of pannexin 1 and 2 in cortical lesions from intractable epilepsy patients with focal cortical dysplasia

SUPPLEMENTARY TABLES

Supplementary Table 1: Clinical and neuropathological features of patients with FCD

See Supplementary File 1

Supplementary Table 2: Clinical and neuropathological features of autopsy subjects

Subjiect No.	Gender	Pathology	Age at autopsy	PMI	Cause of death	Brain region	Application in present study
1	F	Normal	1.5	2.0	Choking/suffocation	F,P,O	Real-time PCR,WB,IHC
2	M	Normal	2.2	1.5	Drowning	F,T	Real-time PCR, WB,IHC
3	M	Normal	2.6	0.5	Organophosphorus poisoning	T,O	WB,IHC
4	M	Normal	4.3	5.5	Drowning	F,P,O,T	Real-time PCR, WB,IHC
5	F	Normal	6.2	3.5	Drowning	F,P	Real-time PCR, WB,IHC
6	M	Normal	7.1	1.5	Non-neurological disease	F,T,P	Real-time PCR, WB,IHC
7	F	Normal	8.2	2.5	Motor vehicle accident	F	WB,IHC
8	M	Normal	8.5	3.0	Drowning	T,P,O	Real-time PCR, WB,IHC
9	M	Normal	10.4	2	Drowning	F,O	Real-time PCR,WB,IHC
10	M	Normal	10.8	2.5	Motor vehicle accident	F,T	Real-time PCR, WB,IHC

PMI, Post-mortem interval (the interval between death of one patient and removal of the brain prior to freezing or fixation); F, female; M, male; F, frontal lobe; P, parietal lobe; O, occipital lobe; T, temporal lobe; WB, western blotting; IHC, immunohistochemistry (including immunofluorescence).
