# Supplementary material for: Expression of pannexin 1 and 2 in cortical lesions from intractable epilepsy patients with focal cortical dysplasia
Source: Oncotarget. 2016 Dec 28;8(4):6883–95. doi: 10.18632/oncotarget.14317 (PMC5351677; doi:10.18632/oncotarget.14317)
Supplement: Supplementary file 2 [file oncotarget-08-6883-s002.docx]

Supplemental Table 1. Clinical and neuropathological features of patients with FCD

| Case No. | Gender | Pathology | Age at surgery (years) | Epilepsy  duration (years) | Seizure  type | | Epileptogenic lesions  location | Seizure frequency  (per month) | AEDs | PO | Application in present study |
| --- | --- | --- | --- | --- | --- | --- | --- | --- | --- | --- | --- |
| 1 | F | FCDIa | 2.6 | 1.9 | IS | | T, P | 115 | VPA, ACTH  LEV, CLZ | I | Real-time PCR, WB, IHC |
| 2 | M | FCDIa | 4.6 | 3 | PS, GTCS, Tonic | | T, P | 25 | OxCZ,LEV, LMT | III | Real-time PCR, WB,IHC |
| 3 | M | FCDIa | 5.0 | 2.5 | PS, Tonic | | F | 60 | CBZ, VPA, PB | II | Real-time PCR, WB,IHC |
| 4 | F | FCDIa | 5.5 | 3.0 | PS, GTCS | | F, T | 15 | CBZ, NP, VPA, LMT, CLZ | II | Real-time PCR, WB,IHC |
| 5 | M | FCDIa | 6.0 | 4.5 | PS, GTCS | | F | 45 | CBZ, VPA, LMT | I | IHC |
| 6 | F | FCDIa | 6.5 | 3.0 | PS | | T | 45 | OxCZ, LMT, TPM, VPA | I | IHC |
| 7 | M | FCDIa | 7.0 | 4.0 | PS | | F | 60 | CBZ, LEV  OxCZ, LMT | I | Real-time PCR, WB,IHC |
| 8 | M | FCDIa | 9.0 | 3.0 | PS | | T | 75 | OxCZ, LEV  CLZ, VPA | IV | IHC |
| 9 | F | FCDIa | 9.6 | 6.5 | GTCS | | O | 25 | VPA, LEV, TPM | I | Real-time PCR, WB,IHC |
| 10 | M | FCDIa | 11.0 | 6.0 | GTCS, Tonic | | F | 13 | VPA, LEV, CLZ | III | Real-time PCR, WB,IHC |
| 11 | F | FCDIa | 12.0 | 5.5 | PS, GTCS | | T | 100 | PHT, CBZ, VPA,  LEV, TPM, CLZ | II | Real-time PCR, WB,IHC |
| 12 | F | FCDIIa | 1.2 | 1.0 | PS, Tonic | | P; O | 35 | VPA, OxCZ,LEV | I | IHC |
| 13 | F | FCDIIa | 2.5 | 2.0 | IS | | F | 45 | LEV, ACTH, GC, VPA, NP, CLZ | I | Real-time PCR, WB,IHC |
| 14 | M | FCDIIa | 3 | 2.5 | GTCS | | F, T | 10 | VPA, LEV, TPM | IV | IHC |
| 15 | F | FCDIIa | 4.2 | 4.0 | IS | | F, P | 15 | VPA, ACTH, GC, CLZ, LMT | I | IHC |
| 16 | M | FCDIIa | 5.6 | 3.0 | PS, GTCS, IS | | F | 105 | CBZ, LEV, VPA, GC, CLZ | II | Real-time PCR, WB,IHC |
| 17 | M | FCDIIa | 6.5 | 5.0 | GTCS | | F | 10 | VPA, LEV, TPM | I | Real-time PCR, WB,IHC |
| 18 | F | FCDIIa | 7 | 6.5 | PS, Tonic | | P | 125 | OxCZ, VPA,LMT | III | Real-time PCR, WB,IHC |
| 19 | F | FCDIIa | 7.5 | 6.0 | PS, GTCS | | T | 20 | LEV, TPM, VPA, OxCZ, LMT | I | Real-time PCR, WB,IHC |
| 20 | M | FCDIIa | 8 | 6.5 | PS, GTCS | | F, O | 55 | VPA, CBZ, CLZ | II | Real-time PCR, WB,IHC |
| 21 | M | FCDIIa | 9.5 | 8.0 | PS | | F | 205 | OxCZ, LMT, VPA, TPM, NP | I | Real-time PCR, WB,IHC |
| 22 | F | FCDIIa | 10.2 | 8.0 | PS, Tonic | | F | 25 | TPM,CBZ | I | IHC |
| 23 | M | FCDIIa | 11.5 | 9.5 | GTCS | | T | 8 | VPA,CLZ | I | Real-time PCR, WB,IHC |
| 24 | F | FCDIIb | 1.6 | 1.0 | GTCS | T | | 15 | PHT,CBZ,PB | I | Real-time PCR, WB, IHC |
| 25 | M | FCDIIb | 2.0 | 1.5 | PS, IS | F | | 45 | TPM,PHT,ACTH | I | IHC |
| 26 | M | FCDIIb | 3.8 | 2.2 | PS | F | | 105 | VPA,TPM,LEV, LMT OxCZ, | I | Real-time PCR, WB, IHC |
| 27 | M | FCDIIb | 4.0 | 2.3 | PS,GTCS | F | | 240 | OxCZ,VPA,LEV,  NP,TPM | I | IHC |
| 28 | F | FCDIIb | 4.3 | 3.3 | PS,GTCS | P | | 18 | VPA,LMT | II | Real-time PCR, WB, IHC |
| 29 | F | FCDIIb | 5.5 | 5.0 | GTCS, IS | F,T | | 135 | CBZ,VPA, ACTH,GC | I | Real-time PCR, WB, IHC |
| 30 | F | FCDIIb | 5.5 | 4.1 | GTCS, Tonic | T | | 12 | VPA,TPM,  CBZ,LEV | III | Real-time PCR, WB, IHC |
| 31 | M | FCDIIb | 6.3 | 4.5 | PS, IS | T | | 120 | VPA, PB, LCZ  ACTH, GC | II | Real-time PCR, WB, IHC |
| 32 | F | FCDIIb | 7.5 | 4.8 | PS, GTCS, Tonic | F | | 25 | VPA,LEV | III | Real-time PCR, WB, IHC |
| 33 | F | FCDIIb | 9.2 | 7.5 | PS | O | | 26 | VPA,CLZ,TPM | I | Real-time PCR, WB, IHC |

FCD, focal cortical dysplasia; AEDs, antiepileptic drugs; PO, postoperative outcome (Engel’s class); F, female; M, male; IS, infantile spasm; PS, partial seizure; GTCS, generalized tonic-clonic seizure; F, frontal lobe; P, parietal lobe; O, occipital lobe; T, temporal lobe; PHT, phenytoin; CBZ, carbamazepine; PB, phenobarbital; TPM, topiramate; ACTH, adrenocorticotropic hormone; GC, glucocorticoid; VPA, valproate; OxCZ, oxcarbazepine; LEV, levetiracetam; LMT, lamotrigine; CLZ, clonazepam; NP, nitrazepam; WB, western blotting; IHC, immunohistochemistry (including immunofluorescence).
